# A randomized, double-blinded, placebo-controlled clinical trial of duloxetine hydrochloride enteric-coated tablets in the treatment of refractory chronic cough

**DOI:** 10.1186/s12890-023-02575-5

**Published:** 2023-08-02

**Authors:** Shengyuan Wang, Shaohui Li, Heng Wu, Tongyangzi Zhang, Yixiao Chen, Yiqing Zhu, Siwan Wen, Cuiqin Shi, Li Yu, Xianghuai Xu

**Affiliations:** 1 Department of Pulmonary and Critical Care Medicine, School of Medicine, Tongji Hospital, Tongji University, No. 389 Xincun Road, Shanghai, 200065 China; 2 Department of Otorhinolaryngology - Head and Neck Surgery, School of Medicine, Tongji Hospital, Tongji University, No. 389 Xincun Road, Shanghai, 200065 China; 3 Department of Psychosomatic Medicine, School of Medicine, Tongji Hospital, Tongji University, Shanghai, 200065 China

**Keywords:** Duloxetine, Cough, Prospective studies

## Abstract

**Introduction:**

Refractory cough, a chronic cough with an unclear diagnosis or poor treatment response. The symptoms are often stubborn and persistent, causing serious complications and lowering the patient's quality of life. Cough hypersensitivity syndrome (CHS) is proposed as a potential cause, and reducing sensory nerve hyperresponsiveness is suggested as an effective treatment. However, current drugs have low efficacy and benefit rates and numerous side effects. This trail proposes using duloxetine, a selective 5-HT and norepinephrine reuptake inhibitor, as a potential treatment for refractory cough, which has shown promise in treating pain and depression. Duloxetine may inhibit pain conduction and oxidative stress in peripheral nerves by inhibiting the activity of TRPV1 channels, which play an important role in the peripheral afferent pathway of refractory cough. Meanwhile, the antidepressant effects of duloxetine may also play a role in the treatment of refractory cough.

**Methods and analysis:**

This is a single-center, prospective, randomized, double-blind, and controlled trial. A total of 98 individuals will be randomized in a 1:1 ratio to duloxetine group and placebo control group (starting with 20 mg QD, increasing 20 mg daily until 20 mg TID). After a screening period, the second stage runs from baseline to the 42nd (last) day of treatment, with follow-up visits on the 3rd, 7th, 14th, 21st, 28th, 35th, 42nd and 49th days. The main end-stage observation indicators include objective cough frequency, cough visual analog scale (VAS), cough symptom score, Leicester Cough Questionnaire (LCQ), and cough evaluation test (CET); the secondary end-stage observation indicators include capsaicin cough sensitivity, Patient Health Questionnaire-9 (PHQ-9), Major Depression Inventory (MDI), the Generalized Anxiety Disorder-7 scale (GAD-7), Life Events Scale (LES-32), induced sputum supernatant. The safety measures will be AEs/SAEs, vital signs, liver and kidney function, fecal occult blood test.

**Discussion:**

This study is the first randomized, double-blind, and controlled clinical trial investigating the use of duloxetine in the treatment of refractory coughs. The study aims to provide a high-quality basis for evaluating the efficacy and safety of duloxetine for this condition.

**Trial registration:**

Our study was registered in the Chinese Clinical Trials Register (www.chictr.org.cn/) (ChiCTR2000037429) in 28/08/2020.

**Supplementary Information:**

The online version contains supplementary material available at 10.1186/s12890-023-02575-5.

## Background

Refractory cough is a chronic cough with unclear diagnosis or with a definite diagnosis but poor curative effect. The diagnosis and treatment process recommended by the existing cough guidelines requires that on the basis of careful medical history inquiry and physical examination, necessary auxiliary examinations be selected to identify the cause of chronic cough, and the treatment response to the cause should be determined to confirm or exclude the diagnosis. Sometimes, even after a comprehensive auxiliary examination, the cause of cough cannot be identified, or although the cause can be identified, there is no effective treatment or no good treatment response, resulting in stubborn or persistent cough symptoms. All of the above are refractory coughs [1]. The proportion of refractory cough among cases of chronic cough varies between regions, up to 42%, and our department has found rates of 5–12% [2, 3]. The symptoms of patients with refractory cough are often stubborn and persistent. Severe coughing not only causes headache, chest pain, vomiting, difficulty sleeping, fatigue, cough syncope, urinary incontinence, and other manifestations, as well as complications such as rib fractures, but also makes patients irritable, depressed, and anxious, which seriously lower their quality of life [4, 5]. These patients often seek medical treatment in multiple hospitals, spend huge amounts of money, undergo repeated auxiliary examinations, take various therapeutic drugs indiscriminately, and suffer the risks of drug side effects, causing serious social problems and economic burdens [6].

The cough sensitivity of patients with chronic cough is significantly higher than that of normal people, and it has obvious homology with the basic neurobiological mechanism and pathology of chronic pain [7, 8]. Based on this, Morice et al. proposed the concept of cough hypersensitivity syndrome (CHS), which is defined as a type of disease with chronic cough as the only or most prominent symptom on the basis of cough hypersensitivity [9]. Based on this concept, modulating cough sensitivity has become an important strategy for chronic cough treatment [10, 11]. Much like the mechanism of pain, the occurrence of CHS involves neurogenic inflammation, the transient receptor potential (TRP) family, and other factors. A growing number of researchers believe that both can be caused by sensory hyperactivity (SHR), called hypersensitivity syndrome or neuropathic disease [8]. Appropriately reducing sensory nerve hyperresponsiveness could theoretically be an effective treatment. Neuromodulatory drugs such as gabapentin and P2X3 antagonists, as well as nonpharmacological cough suppressant therapy, have shown a certain effect in reducing cough sensitivity and cough frequency [12–14]. However, the above drugs not only have low efficacy and benefit rates but also have many side effects, such as drowsiness, dizziness, and dysgeusia. Transient receptor potential vanilloid 1 (TRPV1) and transient receptor potential channel subfamily A member 1 (TRPA1) receptor blockers, which have given high hopes to some, have not shown therapeutic effects in recent clinical studies on refractory cough [15, 16] Therefore, patients with refractory cough have few recourses for treatment.

As a selective 5-hydroxytryptamine (5-HT) and norepinephrine (NE) reuptake inhibitor, duloxetine is a strong neuronal 5-HT and NE reuptake inhibitor that has a relatively weak inhibitory effect on dopamine reuptake. It is well tolerated, has few adverse reactions, and has been widely used in pain treatment [17, 18]. *N*-methyl-d-aspartate (NMDA) receptor, gamma-aminobutyric acid (GABA), 5-HT, and NE play important roles in the development of cough and pain and also play relevant roles in the formation of cough hypersensitivity [19, 20]. Theoretically, dual 5-HT and NE reuptake inhibitors could suppress central hypersensitivity by impairing nociceptive pain uploading [21] and could be used in the treatment of refractory cough. Jeyakumar et al. used low-dose amitriptyline to treat refractory cough secondary to vagal pathology induced by viral infection. After 10 days of treatment, the cough frequency, severity, and quality of life of the patients were significantly improved, but most patients stopped treatment due to its side effects, of which the most common were sedation and dry mouth, followed by anxiety, insomnia, dizziness, and weight gain [22]. Duloxetine has no affinity for adrenergic receptors, cholinergic receptors, or histamine receptors, so it causes none of the adverse effects after blockade of α1 and H1 receptors that are common with tricyclic antidepressants such as amitriptyline, giving it good safety and tolerance [23]. The U.S. Food and Drug Administration has approved duloxetine for the treatment of diabetic peripheral neuralgia, fibromyalgia, and chronic musculoskeletal pain. Duloxetine hydrochloride is as effective as traditional antidepressants but is more effective faster and has fewer adverse reactions [24]. Therefore, the analgesic effect of duloxetine may also be applied to the improvement of cough symptoms. Duloxetine may inhibit pain conduction and oxidative stress in peripheral nerves by inhibiting the activity of TRPV1 channels [25], while TRPV1 also plays an important role in the peripheral afferent pathway of refractory cough [26]. Therefore, duloxetine may also reduce peripheral nerve sensitivity in patients with refractory cough by inhibiting the activity of TRPV1 channels.

In addition to possibly improving cough symptoms, the antidepressant effects of 5-HT and NE reuptake inhibitors may also play a role in the treatment of refractory cough. Psychological symptoms are interrelated with cough severity and quality of life. Chronic cough seriously affects the quality of life of patients, causing fatigue, sleep deprivation, and social withdrawal, leading to increased complications of psychiatric disorders such as anxiety and depression. These psychological problems may affect their perception of symptoms and are associated with decreased treatment adherence and slower response to treatment. French et al. found that symptoms of depression, anxiety, and stress also gradually improved when cough symptoms were relieved. They believed that psychological factors, symptom experience, and daily functions interacted and influenced each other, so the authors proposed that intervention on the psychological state may also help improve somatic symptoms and quality of life [27]. As a new type of antidepressant, duloxetine has been used in the treatment of depression and generalized anxiety disorder. It has also shown efficacy in patients with somatic symptom disorders, significantly reducing the level of somatization and functional disability [28]. Therefore, duloxetine can reduce the psychological burden of patients and help improve the symptoms of refractory cough. We propose this study to test the hypothesis that duloxetine has a good therapeutic effect on patients with refractory cough.

### Research objective

The primary objective of this study is to investigate whether duloxetine can improve symptoms in patients with refractory chronic cough.

### Research methods

Through a randomized, double-blinded, placebo-controlled clinical trial, we will explore whether duloxetine hydrochloride enteric-coated tablets can improve the cough symptoms of refractory cough and its underlying mechanisms.

### Research content and methods

(1) We will compare the starting time and effectiveness rate of duloxetine hydrochloride enteric-coated tablets vs. placebo for the reduction of cough symptoms and somatic symptoms in patients with refractory cough. We will also observe whether duloxetine hydrochloride enteric-coated tablets can relieve or control cough symptoms in patients. (2) After discontinuation of duloxetine hydrochloride enteric-coated tablets, the change pattern and effectiveness rate against cough and somatic symptoms of the two groups of patients will be compared, and will determined whether there is a continuation effect of duloxetine hydrochloride enteric-coated tablets after drug discontinuation.

(3) We will compare the changes on the objective cough frequency, cough visual analog scale (VAS), cough symptom score, Leicester Cough Questionnaire (LCQ), cough evaluation test (CET), capsaicin cough sensitivity, Patient Health Questionnaire-9 (PHQ-9), Major Depression Inventory (MDI), the Generalized Anxiety Disorder-7 scale (GAD-7), Life Events Scale (LES-32), and induced sputum supernatant at each time point to explore whether duloxetine acid enteric-coated tablets further improve the quality of life and cough sensitivity of patients with refractory cough.

(4) We will compare the drop-out rate and the completion rate of the treatment course in the two groups of patients to determine whether duloxetine hydrochloride enteric-coated tablets can improve the treatment adherence and success rate of patients.

The first stage is a screening period of up to 3 days and a lead-in period of up to 4 days. The subjects who sign the informed consent form (ICF) will screened, and the qualified subjects will enter the lead-in period. The second stage runs from baseline to the 42nd (last) day of treatment, with follow-up visits on the 3rd, 7th, 14th, 21st, 28th, 35th, and 42nd days to observe the efficacy and safety of the test drugs. The third stage is the follow-up 1 week after treatment ends.

### Inclusion criteria

All patients included in the study must meet all of the following criteria:

- ① Patients or their legal representatives must sign an ICF before any assessment;
- ② 18 years ≤ age ≤ 60 years, male or female;
- ③ Patients with chronic cough whose main symptom is cough, whose cough has lasted for more than 8 weeks, and who have no obvious abnormality in the frontal or lateral chest X-ray. A comprehensive examination according to the diagnostic procedures of chronic cough recommended by China's *Guidelines for Diagnosis and Treatment of Cough (2021)* still cannot identify the cause, or the patient has had no relief despite targeted treatment for known causes.
- ④ Subjects with a cough VAS ≥ 30 mm;

### Exclusion criteria

Screened patients who meet one of the following criteria are not eligible for the study:

- ① Pregnant, breastfeeding women or those who refuse to sign the ICF;
- ② Smoking or having quit smoking ≤ 2 years;
- ③ History of respiratory tract infection within 8 weeks;
- ④ Renal insufficiency, pregnant women, breastfeeding women;
- ⑤ Patients who need to take an angiotensin-converting enzyme inhibitor (ACEI) for a long time;
- ⑥ Other psychiatric disorders or drug dependence;
- ⑦ Patients with hypertension or other cardiovascular system diseases;
- ⑧ The researcher believes that there are other circumstances that make the patient unsuitable for entry into the study.

### Sample size estimation

According to our previous treatment of 11 patients diagnosed with refractory cough with duloxetine hydrochloride enteric-coated tablets at 20 mg TID, seven patients had more than 50% relief from the their cough, and some patients even enjoyed full relief of their cough. The remission rate was 63%. Placebo was 30% effective for refractory cough. According to the estimation formula of the sample size for the comparison of the two sample rates, a two-sided test will be performed at the level of significance of 0.05 and the level of statistical power of 0.8. The number of observed cases needed in each group is 42, so with an estimated loss to follow-up rate of 15%, the number of cases to be observed in each group is 49. A total of 98 cases of refractory cough which seek medical treatment in Tongji Hospital, School of Medicine are needed in the study to detect true results at a statistical significance level of 5%.

### Randomization

According to the random number method set by a computer, each patient is divided into a duloxetine group and a placebo control group.

### Blinding

The trial will be arranged and controlled by the research designer from the aspects of random number generation, allocation of experimental drug codes, experimental drug codes, registration of subjects, recording and evaluation of trial results, research monitoring, and data management. Both investigators and the subjects will be blinded. For the smooth implementation of the double-blinded trial, double-blinded simulation technology will be used to ensure that the provided placebo is identical to the actual duloxetine capsules in terms of dosage form, appearance, properties, smell, etc., but do not contain active ingredients. Both duloxetine capsules and placebo will be labeled with the name "duloxetine," along with the dosage, storage method, and drug number. Unblinding should be done only if the subject has a significant adverse event (SAE).

### Interventions

At baseline, subjects with refractory cough who meet the inclusion and randomization criteria of this study and do not meet the exclusion criteria will be randomly assigned to the experimental drug group or the placebo group, and the experimental group will be given duloxetine hydrochloride enteric-coated tablets 20 mg, TID (starting with 20 mg QD, increasing 20 mg daily until 20 mg TID). The placebo group will receive placebo 20 mg, TID (started with 20 mg QD, increased 20 mg daily until 20 mg TID).

### Criteria for test drug reduction or discontinuation

Reduction or discontinuation of the test drug should be avoided during the trial. However, the investigator will consider dose reduction, suspension, or discontinuation if one of the following occurs:

- ① Occurrence of SAEs related to the test drug;
- ② Adverse reaction (AR) to the tested drugs that cannot be effectively relieved by symptomatic treatment;
- ③ Complications/new onset of other diseases that the test drug may aggravate;
- ④ Significant abnormalities in safety-related laboratory indicators;
- ⑤ Other situations that make investigators believe that dose reduction, suspension, or discontinuation is warranted.

### Combination medication

No combination medication will be tested.

### Prohibited concomitant medication

We will try to avoid combining drugs as much as possible, especially combining medications that have a strong impact on the judgment of the trial results (effectiveness and safety). However, in case of AE, deterioration of the original condition, or complication/new onset of other serious diseases during the trial, the investigator should promptly combine the drugs and actively carry out rescue treatment. If the subject meets the withdrawal criteria, the investigator should make proper arrangements for the subject to withdraw from the trial. All the concomitant medication (including prescription and over-the-counter medications) should be recorded in the case record form (CRF) during the trial, including details of drug name, single dose, frequency of administration, routine of administration, reason for administration, start and end time of administration, etc. If the dosage of concomitant medication changes, it should also be recorded in the CRF. In particular, the combined drug use in the event of an AE should be recorded and reported in a timely way.

### Subject elimination criteria

Subjects can voluntarily withdraw from the trial at any time for any reason, and the investigator can also suspend any subject from participating in the trial for various reasons (mainly including safety issues or breach of protocol). Subjects who have one of the following circumstances shall withdraw from the trial or be deemed to have withdrawn from the trial:

- ① The subjects stopped taking the drug on their own and were unwilling to continue the trial due to poor efficacy or ineffectiveness;
- ② Subjects withdraw their ICF and voluntarily withdraw from the trial;
- ③ Subjects are lost to follow-up.

### SAE or more severe allergic reactions:

- ① The subjects are not allowed to continue using the experimental drugs if the investigator and/or monitor thinks it is unsafe and unethical. This is because of the significant abnormalities in safety-related laboratory indicators, the occurrence of SAEs, or more severe allergic reactions associated with the study drug;
- ② The patient goes blind due to SAEs or needs to be resuscitated immediately;
- ③ The patient gets pregnant during the trial;
- ④ It is inappropriate for the patient to continue participating in the trial due to the worsening of the original disease or lack of tolerance during the trial;
- ⑤ If the trial results in complications or the onset of new serious illnesses, such as tuberculosis or tumors, the investigator will deem it necessary to discontinue the trial.

### Serious violation of the test procedure

- ① Due to serious violations by the investigator, such as the enrollment of patients who do not meet the inclusion criteria and/or who meet the exclusion criteria, or subjects who do not sign the ICF;
- ② Due to serious violations of the subjects, such as failure to complete the test items stipulated in the protocol, meaning the evaluation cannot be carried out, or unintended pregnancy during the trial.

### Test exit process

- ① If the patient signs the ICF but is not randomized, the investigator will document the patient's demographic characteristics and reasons for withdrawal and whether AEs or SAEs occurred during the presentation period.
- ② If the subject withdraws from the trial halfway, the investigator should try to complete the inspection and evaluation of various indicators within 2 weeks after withdrawal (preferably before the start of other treatments) as the final follow-up measures. Investigators should contact patients lost to follow-up. For patients who terminate the trial early, the date of the last dose and the reason for early termination should be recorded in the CRF.

Subjects who withdraw from the trial after randomization cannot be replaced. Each randomization number is uniquely associated with each subject and cannot be reused.

### Effectiveness evaluation

Clinical data will be recorded during the lead-in period before randomization. The objective cough frequency, cough VAS, cough symptom score, LCQ, capsaicin cough sensitivity, PHQ-9, MDI, GAD-7 scale, and LES-32 will be evaluated and recorded, and induced sputum supernatant will be collected. During the treatment period, the cough VAS score will be recorded every day, and the questionnaire will be recovered after the treatment. After randomization, the subjects will be followed up by telephone once on the 3rd and 7th days to evaluate and record the patients' cough VAS score, daytime score, nighttime score, CET score and adverse events (AEs). On the 14th, 21st, 28th, 35th, and 42nd days, the patients will return to the center to evaluate and record their cough VAS, daytime score, nighttime score, CET score, LCQ, MDI, GAD-7, LES- 32 and cough frequency, capsaicin cough sensitivity and induced sputum supernatant will be recorded on the 14th and 42nd days.

**Fig. 1:**
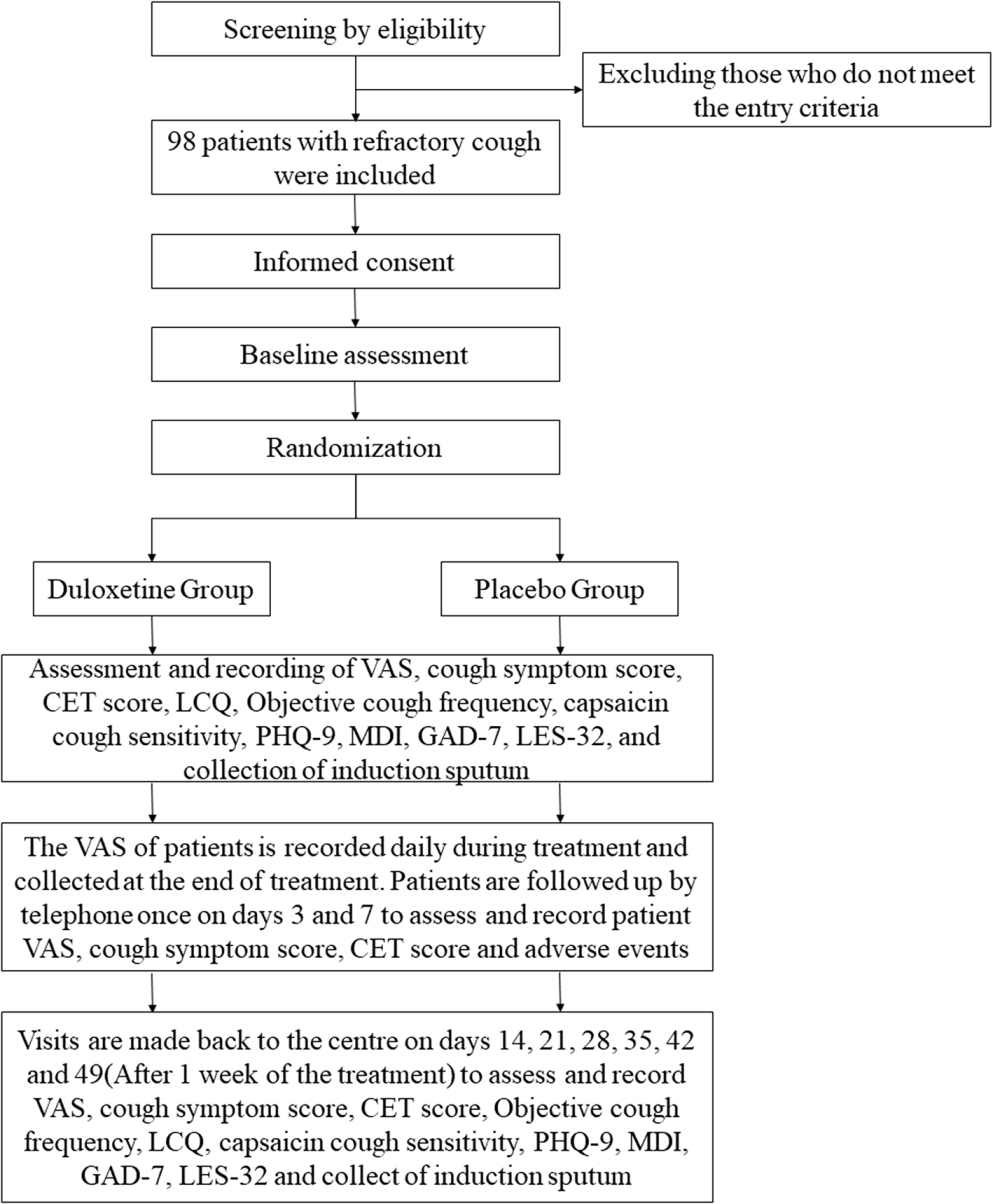
The flow of patients through the study. VAS, visual analog scale; LCQ, Leicester Cough Questionnaire; CET, cough evaluation test; PHQ-9, Patient Health Questionnaire-9; MDI, Major Depression Inventory; GAD-7, the Generalized Anxiety Disorder-7 scale; LES-32, Life Events Scale

One week after the completion of the treatment period, the patients are followed up again to evaluate and record the cough frequency, cough VAS, daytime score, nighttime score, CET score, LCQ, capsaicin cough sensitivity, PHQ-9, MDI, GAD-7, LES-32, and collect induced sputum supernatant. After the follow-up, the blinding will be unmasked, and the patients in the control group will be treated with duloxetine hydrochloride enteric-coated tablets 20 mg TID (started with 20 mg QD, increased 20 mg daily until 20 mg TID) for 42 days. The schedule of events for the enrolment, interventions and assessments for participants is shown in Fig. 1 and Table 1.

**Table 1:** Outline of planned visit

Timepoint	Study period									
	Screening visit	Randomize	Treatment visits							Post-treatment follow-up
	−7 days	Day 0	Visit 1(phone interview)	Visit 2(phone interview)	Visit 3	Visit 4	Visit 5	Visit 6	Visit 7	Visit 8
			Day 3	Week 1	Week 2	Week 3	Week 4	Week 5	Week 6	Week 7
Eligibility screen	✔									
Informed consent	✔									
Adverse event check			✔	✔	✔	✔	✔	✔	✔	✔
VAS			✔	✔	✔	✔	✔	✔	✔	✔
Cough symptom score	✔		✔	✔	✔	✔	✔	✔	✔	✔
Objective cough frequency	✔				✔	✔	✔	✔	✔	✔
CET	✔		✔	✔	✔	✔	✔	✔	✔	✔
LCQ	✔				✔	✔	✔	✔	✔	✔
PHQ-9	✔				✔	✔	✔	✔	✔	✔
MDI	✔				✔	✔	✔	✔	✔	✔
GAD-7	✔				✔	✔	✔	✔	✔	✔
LES-32	✔				✔	✔	✔	✔	✔	✔
Induction sputum	✔				✔	✔	✔	✔	✔	✔
Capsaicin cough sensitivity	✔				✔	✔	✔	✔	✔	✔

Notes: *VAS* visual analog scale, *LCQ* Leicester Cough Questionnaire, *CET* cough evaluation test, *PHQ-9* Patient Health Questionnaire-9, *MDI* Major Depression Inventory, *GAD-7* the Generalized Anxiety Disorder-7 scale, *LES-32* Life Events Scale

### Data analysis

The data are input into the computer by a dedicated person. After all studies are completed, unblinding and subsequent statistical processing will be carried out according to standard procedures. The data with homogeneous variances will be expressed as mean ± standard deviation, and the data with heterogeneous variances will be expressed as median (interquartile spread). The chi-squared test will be used for count data, and the t-test will be used for measurement data. All participants who remained eligible at their initial brochure mailing date will be analyzed according to the group they were originally assigned. Missing data will be closely tracked.

### Patient and public involvement

Patients will not be involved in this study.

## Result

### Main end-stage observation indicators

Main end-stage observation indicators include cough frequency, cough VAS, cough symptom score, LCQs, and CET.

### Secondary end-stage observation indicators and safety measures

Secondary end-stage observation indicators include capsaicin cough sensitivity, PHQ-9, MDI, GAD-7, LES-32, induced sputum supernatant. The safety measures will be AEs/SAEs, vital signs, liver and kidney function, fecal occult blood test.

### Criteria for judging efficacy

Recovery: the cough disappears completely; effective: the sum of daytime and nighttime cough symptom scores decreases by 50% or more; ineffective: the sum of daytime and nighttime cough symptom scores decreases by < 50% or increases.

### Adherence assessment

Adherence assessment will be performed. To determine the subject's adherence, which should generally be between 80 and 120% of medication adherence, subjects should return the experimental drug after the treatment period.

Adherence = actual medication dose/theoretical medication dose × 100%.

Actual dose = total amount of medication dispensed—(total amount of remaining return + total amount of lost).

Theoretical dose = the dose of each time × the number of times of the test drug.

Good adherence: 80% to 120%.

### Ethics statement

A randomized, double-blinded, placebo-controlled clinical trial will be undertaken. The research protocol has been reviewed and approved by the Ethics Committee of Shanghai Tongji Hospital (2020-KYSB-160, 2021-086), and it has been registered in the Chinese Clinical Trial Register (registration number ChiCTR2000037429).

### Data governance

This study will adopt paper storage and Microsoft Excel input for preservation and analysis. Authority assignment: Data entry personnel have the authority to input, modify, and resolve questions. Investigators have specific permissions to modify, browse, resolve challenges, and review. Monitors have rights to browse, send/close challenges, freeze data, and lock. Investigators should retain all detailed original documentation of subjects to ensure that the data are accurate, complete, and timely. Original documents and medical records should be clear, detailed, and easily identifiable by those involved in the trial. Research assistants of chief investigating centre are responsible for regular study monitoring.

### Security assessment

Examination of AEs and clinical endpoints will begin with randomization and will continue to focus on individual patients until they complete a 14-week follow-up. At each visit, the investigator or designee will conduct a safety assessment and will specifically review the clinical history associated with the occurrence of AEs or SAEs and their causal relationship to the test drug to assess the severity of the AEs. Details of AEs and clinical events will be recorded in the CRF, regardless whether the AE is related to the test drug. All AEs should be tracked for follow-up until the incident is resolved.

### SAE reporting and AE reporting

AE collection will begin after the patient signs the ICF. All AEs must be recorded in the appropriate section of the CRF whether or not the AE is related to the test drug. All AEs should be described in concise medical terms, including at least the following contents:

- ① name;
- ② start time.
- ③ seriousness;
- ④ measures taken;
- ⑤ progress;
- ⑥ association with the test drug.

In the event of SEAs during the study period, the investigator should report to the State Food and Drug Administration; the health administrative department; the drug administration department of the relevant province, autonomous region, or municipality directly under the Central Government; the ethics committee of the investigator's research center; and the sponsor to study the SAEs within 24 h. The sponsor is responsible for safety inspections throughout the implementation process and ensuring that the research center completes all SAE reports as required by regulatory agencies and local regulations. At the same time, the sponsor must report to the corresponding regulatory agency in accordance with the requirements of regulatory agencies and local laws.

## Discussion

This article describes an exploratory study protocol to investigate the efficacy of duloxetine in patients with refractory cough through a randomized, double-blinded, placebo-controlled clinical trial. Based on the known pathogenesis of refractory cough and the pharmacological mechanism of duloxetine, we hypothesize that duloxetine will relieve the symptoms of refractory cough patients and may further improve their psychological problems, proving itself beneficial to refractory cough patients.

## Supplementary Information

**Additional file 1.**

## Data Availability

Data sharing is not applicable to this article as no datasets were generated or analysed during the current study.

## Abbreviations

CHS: Cough hypersensitivity syndrome
VAS: Visual analog scale
LCQ: Leicester Cough Questionnaire
CET: Cough evaluation test
PHQ-9: Patient Health Questionnaire-9
MDI: Major Depression Inventory
GAD-7: Generalized Anxiety Disorder-7 scale
LES-32: Life Events Scale
TRP: Transient receptor potential
SHR: Sensory hyperactivity
TRPV1: Transient receptor potential vanilloid 1
TRPA1: Transient receptor potential channel subfamily A member 1
5-HT: 5-Hydroxytryptamine
NE: Norepinephrine
NMDA: *N*-Methyl-d-aspartate
GABA: Gamma-aminobutyric acid
ICF: Informed consent form
ACEI: Angiotensin-converting enzyme inhibitor
AEs: Adverse events
SAE: Significant adverse event
CRF: Case record form
